# Ribosomal DNA Copy Number Variation is Coupled with DNA Methylation Changes at the 45S rDNA Locus

**DOI:** 10.1080/15592294.2023.2229203

**Published:** 2023-06-27

**Authors:** Aleem Razzaq, Yosra Bejaoui, Tanvir Alam, Mohamad Saad, Nady El Hajj

**Affiliations:** a College of Health and Life Sciences, Qatar Foundation, Hamad Bin Khalifa University, Doha, Qatar; b College of Science and Engineering, Hamad Bin Khalifa University, Doha, Qatar; c Qatar Computing Research Institute, Hamad Bin Khalifa University, Doha, Qatar

**Keywords:** Ribosomal DNA, Copy number, DNA methylation, Autism spectrum disorder, Schizophrenia, Dosage compensation

## Abstract

The human ribosomal DNA (rDNA) copy number (CN) has been challenging to analyse, and its sequence has been excluded from reference genomes due to its highly repetitive nature. The 45S rDNA locus encodes essential components of the cell, nevertheless rDNA displays high inter-individual CN variation that could influence human health and disease. CN alterations in rDNA have been hypothesized as a possible factor in autism spectrum disorders (ASD) and were shown to be altered in Schizophrenia patients. We tested whether whole-genome bisulphite sequencing can be used to simultaneously quantify rDNA CN and measure DNA methylation at the 45S rDNA locus. Using this approach, we observed high inter-individual variation in rDNA CN, and limited intra-individual copy differences in several post-mortem tissues. Furthermore, we did not observe any significant alterations in rDNA CN or DNA methylation in Autism Spectrum Disorder (ASD) brains in 16 ASD vs 11 control samples. Similarly, no difference was detected when comparing neurons form 28 Schizophrenia (Scz) patients vs 25 controls or oligodendrocytes from 22 Scz samples vs 20 controls. However, our analysis revealed a strong positive correlation between CN and DNA methylation at the 45S rDNA locus in multiple tissues. This was observed in brain and confirmed in small intestine, adipose tissue, and gastric tissue. This should shed light on a possible dosage compensation mechanism that silences additional rDNA copies to ensure homoeostatic regulation of ribosome biogenesis.

## Introduction

In the human genome, the 45S ribosomal DNA array (referred to as rDNA) are clusters of tandemly arranged genes located on multiple acrocentric chromosomes (Chr 13, 14, 15, 21 and 22) that encode the 18S, 5.8S, and 28S subunits of the 45S pre-rRNA transcriptional unit [1–3]. These three rRNA subunits plus the 5S rRNAs subunit along with approximately 70–80 ribosomal proteins are essential components of the eukaryotic ribosome [3]. The 45S rDNA genes are indispensable for human biology since ribosomes are the sites of protein translation and play a role in the maintenance of protein homoeostasis [4]. Therefore, altered ribosome biogenesis and rRNA regulation could influence human health and could be involved in the pathogenesis of several human diseases [5]. For example, changes in ribosome biogenesis are shown to be of vital importance for maintaining pluripotency as well as cell fate determination [6]. Alterations in the structure and function of ribosomes are associated with several human developmental disorders termed ribosomopathies [7]. Furthermore, 45S rDNA copy number (CN) have been reported to have an impact on genome stability and cellular senescence [8,9], whereas rDNA methylation was reported to be associated with ageing and longevity [10–12]. Despite their key role, the 45S rDNA genes were not included in the reference genome assembly because of their repetitive nature and are excluded from studies linking genotype to phenotype [13]. Recently, the v1.0 assembly of the telomere to telomere (T2T) consortium has provided the complete sequences of the short arms of human acrocentric chromosomes using the genome of a hydatidiform mole (CHM13) [14].

The rDNA genes are highly transcribed from the 45S and the 5S rDNA tandem repeat arrays, where rRNA constitutes the vast majority (~80%) of the total cellular RNA [15,16]. The ribosomal DNA arrays also known as the nucleolar organizer regions (NORs) give rise to the nucleolus, a nuclear structure that holds an essential role in ribosome biogenesis and protein translation [17]. Each 45S rDNA repeat consists of an ~ 13 kb ribosomal unit separated by an intergenic spacer region (IGS) ~3 kb long [18]. The coding region is transcribed by RNA polymerase I as a single large RNA (45S pre-rRNA), which is processed to generate the18S, 5.8S, and 28S rRNA subunits [19]. The three coding subunits, 18S, 5.8S, and 28S, are separated by two internal transcribed spacers (ITS1 and ITS2) flanked by the 5´ and 3´ external transcribed spacers (ETS) [20]. The rDNA promoter controls rRNA transcription in this region and is comprised of two distinct cis-control sequences, the core sequence and the upstream control element (UCE) [21]. In humans, the 45S rDNA array shows substantial variability in inter-individual copy number of hundreds of rDNA copies per diploid genome [22]. Obviously, the ribosomal DNA CN determines the amount of ribosomes produced, however only a subset of those copies are transcriptionally active. DNA methylation is reported to regulate the 45S rDNA where inactive rDNA copies are hypermethylated overall, whereas the expressed rDNA copies are mostly hypomethylated [23]. A higher rDNA CN has been postulated to be linked to an increase in the severity of intellectual disability due to a possible increase in synaptic protein synthesis. Similarly, rDNA CN was hypothesized as a possible factor in autism spectrum disorders (ASD) since several of the molecular mechanisms implicated in ASD converge on pathways related to protein synthesis [24]. Recently, rDNA CN has been measured in the Simons Simplex Collection where the authors reported no association of ASD with total number of rDNA copies [25]. The authors measured CN using whole-genome sequencing data derived from blood, however for a better understanding of rDNA aberrations in ASD it would be important to study brain tissue. Similarly, an increase in rDNA CN has been reported in patients with Schizophrenia where measurements were only performed in leukocyte DNA and not in target tissues implicated in Schizophrenia development [26–28]. In addition, it would be valuable to study epigenetic alterations at the 45S rDNA locus in the brains of ASD and Schizophrenia patients since DNA methylation dysregulation has been shown to have an important role in several neurological and developmental disorders as well as in language and communication traits [29–32].

Despite advances in sequencing technologies, accurately quantifying rDNA CN has been challenging due to their highly repetitive nature and length of the repeat units [33]. A few methods have been reported to assess rDNA dosage including droplet digital PCR (ddPCR), contour-clamped homogenous electric field gel electrophoresis (CHEF gels), as well as normalized coverage depth following next-generation sequencing [22,33,34]. More recently, advances in third-generation long-read sequencing such as Pacific Biosciences (PacBio) and Oxford Nanopore have provided a possible avenue for understanding the structure of the rDNA cluster despite the seemingly higher error rate related to the sequencing technology [35,36]. Yet, PacBio sequencing failed to accurately quantify copy number of repetitive DNA including rDNA repeats in Caenorhabditis elegans (*C. elegans*) [37]. To date, whole-genome sequencing via Illumina Sequencing by Synthesis (SBS) technology has been the go-to method for determining rDNA CN in the human genome [22,38,39]. In this study, we tested whether short read whole-genome bisulphite sequencing (WGBS) can be also used to simultaneously assess rDNA CN and DNA methylation. We could determine that rDNA counts calculated using WGBS strongly correlate with those measured using WGS on the same samples, which suggest that WGBS can be also employed to accurately quantify relative rDNA CN. We applied the established pipeline to compare rDNA CN and DNA methylation in ASD brains as well as in neurons and oligodendrocytes from Schizophrenia patients, where we observed no significant differences between cases and controls. Furthermore, we analysed the association between relative rDNA CN and DNA methylation levels, which revealed a strong positive correlation between CN and DNA methylation at the 45S rDNA locus.

## Materials and Methods

In our study, ASD, Schizophrenia, and multi-tissue datasets were analysed. These are publicly available on the NCBI database with BioProject accession numbers as follows: PRJNA321909 (GSE81541) and PRJNA490887 (GSE119981) for ASD; PRJNA421218 and PRJNA422380 for Schizophrenia; and PRJNA34535 for multi-tissue. The 43 kb human rDNA reference sequence was obtained from GenBank (accession: U13369.1) and modified by combining the last 1979 bp with the first 14,000 bp. As such, the rDNA promoter and the 18S, 5.8S, and 28S core elements along with the 5’ and 3’ external transcribed spacer regions (ETS) were included. Regarding Background Read Depth (BRD) calculation, single copy exon and intron reference sequences employed by Gibbons *et al.* and located on acrocentric chromosomes were used [22]. Different filtering steps were performed to obtain this list of single copy exons and introns to be used as a normalized factor. For this purpose, exons with significant BLAST hits (E-value <1× 10–6) to other exons were removed to exclude regions causing ambiguity. Next, only the largest exon was kept from each gene to avoid any mapping to overlapping regions of exons. Finally, only those exons >300 bp were retained to avoid mapping biases. For single copy introns, a similar filtering process was applied along with some additional filters. Introns that show significant BLAST hits (E-value <1× 10–6) to other introns were eliminated to remove ambiguous regions. Only the largest intron was retained to avoid mapping to overlapping regions. Finally, introns >300 bp and less than or equal to 10kb were retained.

## Aligning WGBS reads to rDNA reference sequence

The SRA files were downloaded and converted to FASTQ files using the fastq-dump function of the SRAtoolkit. Adapter contamination and over-represented sequences were removed using Trim Galore (http://www.bioinformatics.babraham.ac.uk/projects/trim_galore/) and reads with a minimum quality score (q) of 20 were retained. Reads whose length is less than 50 nucleotides were discarded and trimmed reads were aligned against the rDNA reference sequence. The WGS reads were aligned against the rDNA sequence using Bowtie2 with default parameters along with the – no mixed and – no discordant options. The output was converted to BAM format and mapped reads were extracted using the samtools ‘view’ command. Next, mapped reads were sorted using the samtools ‘sort’ function and the per base read depth of the 45S rDNA segments (18S and 28S) were extracted using the samtools ‘depth’ command. For the WGBS Neuron and Olig2 libraries, trimmed files were aligned against the reference rDNA sequence using Bismark [40] and the per base depth coverages of rDNA segments were extracted using the samtools ‘depth’ function.

## Sliding window approach

The 45S rDNA segment shows high depth coverage variation along its entire length. In addition, the first 900bp of rDNA 18S shows homology with chr21 and super contig GL000220.17. Therefore, a sliding window of 150bp was applied on the extracted depth coverages of rDNA 18S and 28S of each sample. The CN of 18S and 28S rDNA was then calculated using the selected window.

## Comparing rDNA copy number calculation in WGS and WGBS

The data used to test the rDNA CN calculation pipeline using WGBS data is part of the following BioProject: PRJNA421218 and PRJNA422380 [41,42]. In total, 10 WGS libraries from bulk brain tissue as well as the associated 10 WGBS libraries from each of neurons (NeuN+) and oligodendrocytes (OLIG2+) from the same individuals were used. The genomic DNA was isolated from Brodmann Area 46 (BA46) in all the post-mortem brains. A detailed list of all the studied samples is available in Additional File 1.

## Background read depth (BRD) calculation

The 18S and 28S rDNA CN were normalized using single copy exons and introns located on acrocentric chromosomes *i.e*. background read depth (BRD). To estimate BRD, WGBS and WGS libraries were aligned against the hg19 reference genome using Bismark and bowtie2, respectively. The alignment output was converted to BAM format and sorted based on coordinates using the samtools ‘view’ and ‘sort’ functions. Per base read depth of reference single copy exons and introns were extracted using the samtools ‘depth’ function. The identified single-copy exons and introns located on acrocentric chromosomes (13, 14, 15, 21, and 22) were previously selected by Gibbons et al. after excluding exons and introns with BLAST hits of E < 10–6 to any other region, except for itself, to avoid ambiguous regions [22]. The upper 5% distribution of these depth coverages were removed for each sample to prevent any alignment biases prior to calculating the average read depth (ARD). Since ARD values of exons and introns were close, the Background Read Depth (BRD) was calculated by taking the average of both ARD exons and ARD introns.

## rDNA copy number calculation and validation

The ribosomal DNA CN was calculated using the formula previously employed by Gibbons *et al.* ^*22*^:

rDNA copy number = (ARD of rDNA segments (18S or 28S))/(Background Read Depth (BRD)).

For the validation of rDNA CN calculation using WGBS libraries, a correlation analysis was performed between WGS bulk tissue and WGBS (Neuron and Olig2) libraries CN and the R^2^ and correlation coefficient values were calculated for each region.

## Analysis of schizophrenia and ASD WGBS libraries

Publicly available neuron and oligodendrocyte WGBS libraries were part of the project PRJNA421218 [42]. Overall, 95 WGBS libraries (Neuron = 53 and Olig2 = 42) were analysed to estimate ribosomal DNA CN. Those libraries included 28 Schizophrenia and 25 control neuronal libraries as well as 22 Schizophrenia and 20 control oligodendrocytes. All WGBS libraries were prepared from gDNA isolated from the Brodmann Area 46 (BA46) of the dorsolateral prefrontal cortex. The libraries were trimmed using Trim Galore with default parameters. For relative rDNA CN calculation in ASD, 27 publicly available WGBS libraries including 16 ASD and 11 controls were analysed that were part of the study by Dunaway *et al.* and A Vogel Ciernia *et al.* (GSE81541 and GSE119981) [43,44]. The WGBS libraries were constructed from bulk brain tissue obtained from the Brodmann area 9 (BA9) of the cerebral cortex. Libraries were trimmed using Trim Galore with 7 bp removed from the 5’ end and 10 bp removed from the 3’ end of each library.

## rDNA differential methylation analysis

WGBS trimmed reads were aligned against rDNA reference genome using Bismark [40]. The output bam file was sorted via samtools. Following sorting, an mbias report was generated and bases exhibiting methylation bias at both the 5’ and 3’ end were additionally removed. Methylation calling was performed using the ‘bismark methylation extractor’ tool to extract CpG methylation. Afterwards, the ‘coverage2cytosine’ tool (part of Bismark) was employed with the – merge_CpG option to merge methylation levels of complementary CpG sites from both strands. The output files were used for differential methylation analysis via the DMRseq R package after adjusting for age and gender [45].

## Association between rDNA copy number and DNA methylation

To study the relationship between relative rDNA CN and DNA methylation, the coverage file (.cov) of the rDNA sequence was divided into 200 bp bins (minimum depth coverage of 10 reads with at least 4 CpGs covered) and the average methylation in the 200 bp bins was measured. A linear regression model was applied by regressing rDNA CN of 18S and 28S separately on the calculated average DNA methylation of each of the 200 bp bins. The linear model was adjusted for age, sex, and disease status. Reported p-values were FDR adjusted.

## Effect of depth coverage on rDNA copy number calculation

The aorta libraries used in this analysis are publicly available at PRJNA34535 [46]. After converting the SRA file to FASTQ, adapter trimming was performed via Trim Galore with default parameters (q = 20, length = 50). The trimmed reads were split into 10 files of equal read number using the SeqKit ‘split2’ function. Files were sequentially merged to achieve a depth coverage of 90%, 80%, 70%, 60%, 50%, *etc*. prior to estimating 18S and 28S rDNA CN.

## Results

### Establishing rDNA copy number calculation using WGBS

**Figure 1.**
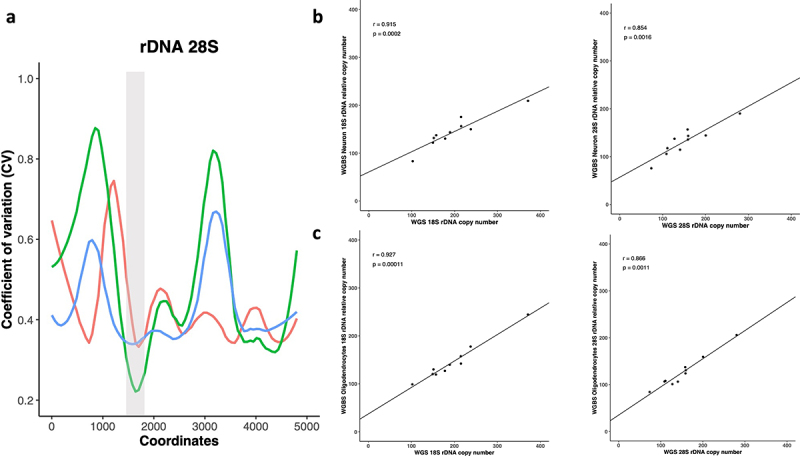
a) a sliding window of 150 bp was applied across the entire 45S rDNA locus where the coefficient of variation (CV) for the depth coverages of all libraries was calculated. The x-axis represents the 28S rDNA coordinates while the CV in depth coverages of WGS brain (red), WGBS Neuron (green) and WGBS Olig2 (blue) is plotted on the Y-axes. The highlighted grey area represents the selected window with minimum CV for 28S rDNA copy number calculation. b) WGS bulk tissue vs WGBS Neuronal libraries for 18S and 28S relative rDNA CN. WGS bulk tissue vs WGBS Oligodendrocyte libraries for c) 18S and 28S relative rDNA CN.

Previously, a computational approach was developed for rDNA CN calculation using average read depth obtained from short-read WGS data [22]. The same approach was also implemented with slight modifications to estimate rDNA CN in tumours [39]. Therefore, we tested whether WGBS data can be also used to estimate rDNA CN count. For this purpose, we employed WGBS and WGS data prepared from the same gDNA that has been extracted from each of 10 individuals. The WGS libraries were performed on bulk brain tissue (BA46), whereas the WGBS data from the same individuals was prepared after separating brain cells into neurons and oligodendrocytes. Per base average read depth of the 18S and 28S rDNA components were extracted and a sliding window was applied on the depth coverages to identify a 150 bp region (in each of 18S and 28S) with the least coefficient of variation across read depth coverages of WGS and WGBS libraries (Figure 1a). The window that showed minimum mean coefficient of variation with a high R-square value across the depth coverages of all samples was selected (18S:6986–7135, 28S:11564–11713).

Next, rDNA CN in the WGS and WGBS libraries were calculated using the selected region, where a correlation analysis between the calculated 18 S rDNA CN of WGS bulk tissue and WGBS neuronal libraries from the same individuals revealed a strong positive correlation (Spearman’s *r* = 0.92, *p* = 0.0002). Similarly, a significant positive correlation was observed between 28S rDNA counts of WGS and 28S WGBS neuronal libraries for the 10 analysed samples (Spearman’s *r* = 0.85, *p* = 0.0016) (Figure 1b,c). Comparing the calculated 18S and 28S rDNA counts for WGS (*N* = 10) vs oligodendrocyte WGBS libraries (*N* = 10) revealed a similarly significant positive correlation for both 18S and 28S rDNA (18S WGS versus 18S WGBS: Spearman’s *r* = 0.93, *p* = 0.00011; 28S WGS versus 28S WGBS: Spearman’s *r* = 0.87, *p* = 0.0011). Furthermore, we observed a strong correlation between the calculated 18S and 28S rDNA CN counts for the neuronal WGBS (Spearman’s *r* = 0.927, *p* = 0.00011) and the oligodendrocyte WGBS libraries (Spearman’s *r* = 0.98, *p* = 1.46^−06^) (Additional File 2). Based on those results, a method to quantify rDNA CN using short read WGBS could be employed to measure relative rDNA copy number.

Afterwards, we tested whether library preparation could have an influence on the calculated rDNA CN. For this purpose, we used two publicly available WGBS libraries prepared from the same sample (aorta tissue) and constructed using two different methods. Here, we observed that the calculated rDNA CN varied substantially between the two different libraries amplified via either PfuTurbo Cx Hotstart DNA Polymerase or the Kapa HiFi Uracil Hotstart polymerase, which is engineered to tolerate Uracil residues. The calculated rDNA CN in the PfuTurbo Cx amplified libraries was 562.62 for 18 S and 521.21 for 28S, whereas the Kapa HiFi library revealed an 18S rDNA CN of 258.28 and a 28S rDNA of 263.19 (Additional File 3). Furthermore, we tested whether depth coverage would have an effect on the calculated rDNA count and DNA methylation levels where we observed that relative rDNA CN and methylation does not vary considerably after decreasing the number of reads (by 10%) starting from total reads down to 10% of the original reads (Additional File 3). Therefore, depth coverage does not seem to substantially affect the calculated copy number, however library preparation method could have a significant influence on rDNA CN calculation.

## Intra-individual rDNA copy number variation in multiple post-mortem tissues

**Figure 2.**
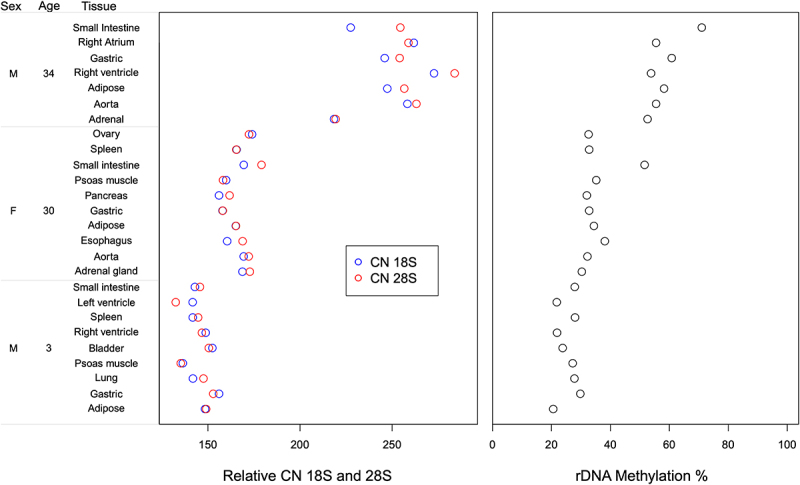
Relative rDNA copy number and global rDNA methylation in multiple post-mortem tissues from each of three individuals.

Using the established pipeline, we estimated relative rDNA CN in post-mortem samples of multiple tissues from three individuals (GSE16256) [46]. In total, nine post-mortem tissues were analysed in individual 1 (age = 3, male), 10 in individual 2 (age = 30, female), and 6 in individual 3 (age = 34, male). This analysis revealed not much variation in the rDNA 18S and 28S relative CN among different tissues within each individual, however we could observe high inter-individual variation in relative rDNA CN between the three studied individuals. In individual 1 (age = 3), the 18S rDNA values ranged between 136 and 156, whereas 28S rDNA count was between 132 and 152 in the 9 studied tissues (Figure 2, Additional File 4). In individual 2 (age = 30), 18S rDNA varied between 156 and 173 copies while 28S rDNA count ranged from 158 to 179. On the other hand, individual 3 (age = 34) exhibited higher relative rDNA CN variation that ranged from 218 to 272 for 18S and 219–283 for 28S. Regarding rDNA methylation, we did not observe major intra-individual differences between the studied post-mortem tissues apart of the small intestine that showed higher rDNA methylation when compared to the remaining tissues in the older individuals. For small intestine, 51.6% methylation was measured in individual 2 vs 33.38% average methylation across all 9 tissues, and 71.00% methylation in individual 3 vs 56.07% average methylation across all 6 analysed tissues (Figure 2, Additional File 4). In individual 1 (3 years old), small intestine rDNA methylation did not vary considerably when compared to other tissues.

## rDNA copy number and methylation in ASD brains

**Figure 3.**
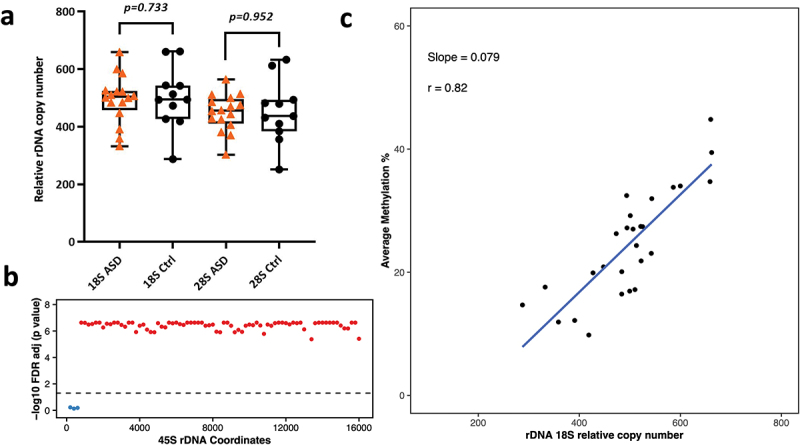
a) Relative 18S and 28S rDNA copy number in ASD vs control brains (16 ASD vs 11 control). No significant difference was observed after adjusting for age and gender. b) Relationship between 18S relative rDNA copy number and DNA methylation at the 45S rDNA locus: 18S rDNA CN was significantly associated with average methylation at the 45S rDNA locus after adjusting for age, gender and disease status. Each dot represents a 200 bp bin whose average DNA methylation was calculated. The obtained p-values were FDR adjusted prior to plotting. The values above the dotted line (red dots) indicate a significant association (*p* < 0.05) while the values below the dotted line (blue) indicate non-significant differences. The x axis represents the 45S rDNA coordinates. c) Scatter plot showing the relationship between relative 18S rDNA copy number and DNA methylation across rDNA coordinates 7400–7599.

Here, we analysed the relative rDNA CN in idiopathic ASD WGBS libraries amplified using PfuTurbo Cx Hotstart DNA Polymerase. In total, 27 publicly available WGBS libraries were analysed including 11 control and 16 ASD brains. The relative 18S rDNA CN in controls ranged from 287 to 661 (average = 501), whereas 28S rDNA CN ranged from 251 to 633 (average = 452). Similarly, the relative rDNA CN in ASD brains was 332–658 for 18S (average = 495) and 302–564 for 28S (average = 449). After adjusting for age and gender using logistic regression, we did not observe any significant differences in rDNA CN in ASD vs control brains (*p* = 0.73 for 18S, *p* = 0.95 for 28S, Figure 3a). We also performed differential DNA methylation analysis on the 45S rDNA locus to determine DNA methylation changes, but no statistical significance was observed after FDR adjustment between ASD and control brains.

## rDNA copy number and methylation in Schizophrenia brains

**Figure 4.**
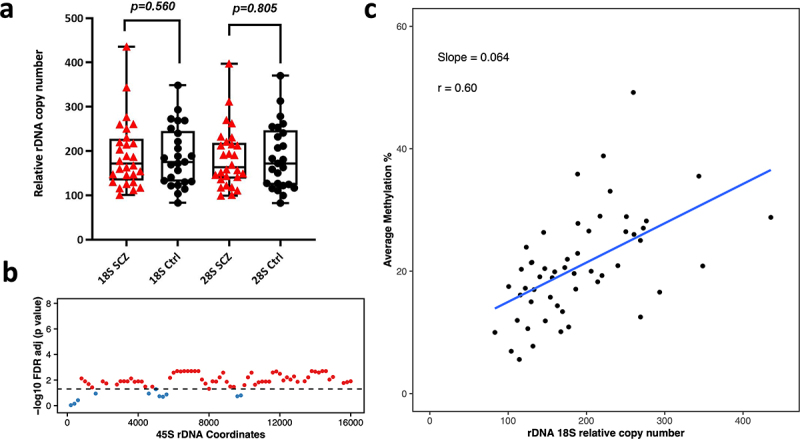
Relative rDNA copy number in a) Schizophrenia (SCZ) neurons vs controls. No significant difference was observed after adjusting for both age and gender. b) Relationship between 18S relative rDNA copy number and DNA methylation at the 45S rDNA locus after adjusting for the following: age, gender and disease status. Each 200 bp bin is displayed as a dot and the average DNA methylation of all CpG sites in the bin was calculated. The obtained p-values were FDR adjusted. The values above the dotted line (red dots) indicate a significant association (*p* < 0.05) while the values below the dotted line (blue) indicate no significant difference. c) Scatter plot for relative 18S rDNA copy number vs DNA methylation across rDNA coordinates 7400–7599 in neurons.

**Figure 5.**
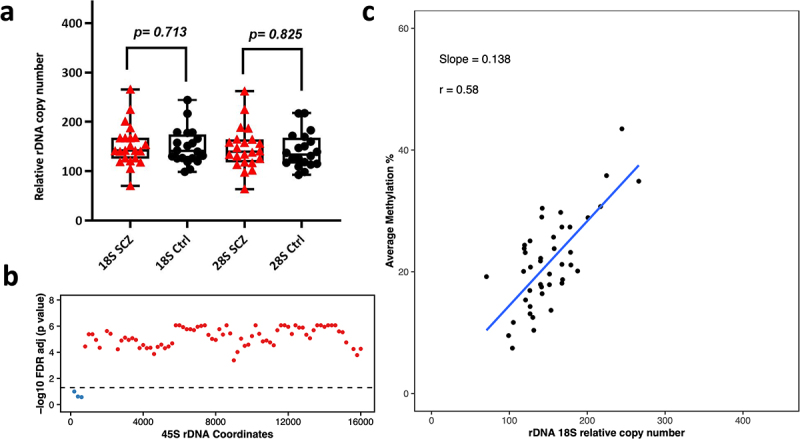
Relative rDNA copy number in a) Schizophrenia (SCZ) oligodendrocytes vs controls. No significant difference was observed after adjusting for age and gender. b) Relationship between 18S relative rDNA copy number and DNA methylation at the 45S rDNA locus in oligodendrocytes after adjusting for age, gender and disease status. Each dot represents a 200 bp bin whose average DNA methylation was calculated. The obtained p-values were FDR adjusted and plotted. The values above the dotted line (red dots) indicate a significant association (*p* < 0.05) while the values below the dotted line (blue) show non-significant differences. The x-axis indicates the 45S rDNA coordinates. c) 18S rDNA copy number vs DNA methylation across rDNA coordinates 7400–7599 in oligodendrocytes.

Next, we analysed 53 Neuronal and 42 Oligodendrocytes WGBS libraries (amplified via Kapa HiFi polymerase) to determine differences in relative rDNA CN and DNA methylation between Schizophrenia patients and controls. These libraries were from the dorsolateral prefrontal cortex region Brodmann Area 46 (BA46). The range of rDNA CN in neuronal control libraries (*N* = 25) was 83–348 copies for 18S (average = 187) and 82–370 for 28S (average = 186). Whereas, the range of 18S rDNA CN in schizophrenia neurons (*n* = 28) was 100–435 (average = 192) and 100–397 for 28S (average = 184). For oligodendrocytes, we observed 98–244 18S copies (average = 149) in control libraries (*N* = 20) and 92–217 for 28S (average = 142). In Schizophrenia libraries (*N* = 22), the 18S rDNA CN ranged from 70 to 266 (average = 152) and the 28S count was 64 to 262 (average = 146). After adjusting for age and gender, we did not observe significant differences in relative rDNA CN of neurons and oligodendrocytes from Schizophrenia patients when compared to controls (SCZ neuron 18S *p* = 0.560, SCZ neuron 28S *p* = 0.805, SCZ Oligodendrocytes 18S *p* = 0.71, SCZ Oligodendrocytes 28S *p* = 0.83, Figures 4a and 5a). However, we could observe a few outliers for rDNA CN in Schizophrenia patients (Figures 4a & 5a). Next, we performed a differential DNA methylation comparison, to compare neurons and oligodendrocytes from Schizophrenia patients vs controls, which revealed no significant DMR with FDR adjusted p-value <0.05.

## Relationship between rDNA copy number variation and DNA methylation levels

We tested the association between relative CN changes and DNA methylation at the 45S rDNA locus. A linear regression model was applied between rDNA CN and average methylation (across 200 bp bins) after adjusting for age, gender, and disease status. The calculated p-values were FDR adjusted and plotted per bin as shown in Figures 3b, 4b, and 5b for 18S (Additional File 5 a, c, e for 28S). Here, we could observe a strong positive correlation between the calculated 18S rDNA CN and average methylation across the majority of the 200 bp bins (apart from the first three) in brain tissue (Figure 3b). Similarly, we could observe a significant positive correlation between relative rDNA CN and DNA methylation for the majority of bins in neurons and oligodendrocytes (Figures 4b and 5b, Additional file 5). Scatter plots for one of the bins spanning coordinates 7400–7599 in bulk brain tissue, oligodendrocytes, and neurons for 18S relative rDNA count vs DNA methylation is displayed in Figures 3c, 4c, and 5c (Additional file 5 b, d, f for 28S). To determine whether this effect is restricted to brain, we compared the relationship between relative rDNA CN variation and global rDNA methylation in the post-mortem multi-tissue libraries. WGBS was performed on three common tissues in the three studied individuals including small intestine, adipose tissue, and gastric tissue. This analysis revealed a positive association between relative rDNA CN and DNA methylation (Additional File 6), indicating that the observed effect is not restricted to brain but also occurs in other tissues.

## Discussion

A pipeline based on short read WGS has been previously established as an accurate method to quantify rDNA CN. In this study, we determined that WGBS could be also used to quantify relative rDNA CN, however the advantages of using WGBS are: 1) the simultaneous quantification of rDNA CN and DNA methylation, and 2) analysis of target tissues involved in disease pathogenesis deposited in public databases (WGS is mainly performed on blood or buccal swab DNA). One important observation is related to drastic differences in calculated rDNA CN when libraries were amplified using PfuTurbo Cx vs Kapa HiFi polymerase, therefore it is important to ensure that similar library preparation methods were employed when comparing samples. The observed effect could be due to amplification bias in certain libraries, which can cause coverage discrepancies in GC and AT rich [47] regions [48]. Due to this bias, we believe that we can only quantify relative but not absolute rDNA CN via WGBS. Sequencing coverage did not have any major effect on quantifying rDNA CN or on the measured DNA methylation levels at the 45 S rDNA locus. Furthermore, we observed a lower estimated copy number in WGBS vs WGS despite the strong correlation between the calculated rDNA CN using both methods. This could be related to the repetitive nature of rDNA where Bismark only retains reads that generate a unique best alignment after running four alignment processes on bisulphite converted reads. It is also worthwhile mentioning that read duplicates were not removed after aligning against the rDNA reference and against the single copy exons and introns, since the 45S rDNA is highly repetitive and rDNA CN quantification could be influenced by depth coverage when read duplicates are removed.

Our analysis on intra-individual differences in rDNA, revealed no major variation in relative CN when looking at several post-mortem human tissues. However, in the older individual, we observed variation of 54 rDNA copies between the analysed tissues, whereas in the remaining two individuals the range was ~20 copies between the tissue with the highest and the one with the lowest number of copies. We could still observe dramatic inter-individual differences between the three studied individuals as well as between the studied brain, neuronal, and oligodendrocyte samples. This is in line with the notion that rDNA CN is not stable and exhibits striking variation among different populations, which has been observed in several species including yeast, drosophila, and mouse [38,49–51]. A handful of studies have also reported alterations in 45S rDNA CN during ageing thus linking biological age to nucleolar biology [52–54]. Nevertheless, results in the literature have been contradictory where Hallgren et al. could not observe rDNA instability in the cerebral cortex of young vs older individuals [55]. The authors could only detect rDNA instability to be associated with neurodegeneration in patients with dementia with Lewy bodies (DLB) [55]. In mice, results on the effect of ageing on 45S rDNA count have been also contradictory where some studies described age-dependent increase in rDNA CN, while other studies reported no differences [56,57].

Our analysis of ASD samples did not reveal major differences at the 45S rDNA locus between ASD and control brains. This is similar to a recently published study performed on blood DNA from individuals enrolled in the Simons Simplex Collection, where ASD patients did not display alterations in rDNA CN [25]. Furthermore, we did not detect significant differences when comparing relative rDNA CN and DNA methylation in neurons and oligodendrocytes from Schizophrenia patients. Previous studies reporting rDNA CN abundance and elevated rRNA gene dosage in Schizophrenia patients included more samples, however rDNA CN was only measured in leukocytes [26–28]. Nevertheless, we could detect a few extreme outliers in the Schizophrenia samples, which might be related to the disease since extremely low or high rDNA CN is known to cause phenotypic variation and lead to adverse health consequences [58].

Our study has also shown that DNA methylation at the 45S rDNA locus is positively associated with relative rDNA CN. This indicates that additional rDNA copies are epigenetically silenced and that DNA methylation might have an influence on the homoeostatic regulation of ribosomal biogenesis. Using NanoPore sequencing, Hori et al. have recently reported that the percentage of methylated rDNA copies increases with higher rDNA CN in lymphoblastoid cell lines from 23 individuals obtained from the Human Pangenomics Project (HPGP) [35]. Similarly, Rodriguez-Algarra et al. reported a significant positive correlation between DNA methylation and total rDNA count in six inbred mouse strains as well as in 48 human lymphoblastoid cell lines [59]. We have observed a similar effect in the studied brain tissues with higher methylation levels across the entire 45S rDNA locus (apart from first 600 bp) in response to increased rDNA CN. Another important insight comes from the multi-tissue analysis, which revealed a similar effect in adipose, gastric, and small intestinal tissue. This shows that the observed effect is not restricted to one particular tissue but systemically affects multiple tissues. A similar dosage compensation mechanism for rRNA transcripts has been recently reported in *Arabidopsis thaliana* following Cas9-mediated genome editing to reduce 45S rDNA CN by ~90% [60]. Despite the drastic reduction in rDNA CN, rRNA levels remained unaffected due to changes in histone marks and altered chromatin organization.

Taken together, we could show that WGBS data could be used to simultaneously measure relative rDNA CN and DNA methylation at the 45S rDNA locus. Neither ASD nor Schizophrenia patients exhibited alterations in relative CN or DNA methylation in brain cells. Our study additionally revealed that rDNA CN variation was coupled with changes in DNA Methylation. Taking into account the effect of rDNA CN on genome stability and cellular senescence, a possible dosage compensation mechanism might control the level of ribosomal RNA transcription in response to variation in rDNA copies. Future work is warranted to understand whether a dosage compensation mechanism regulates ribosomal biogenesis and its implications for cellular function and human health.

## Supplementary Material

Supplemental Material

## Data Availability

The datasets used and analysed in this study are publicly available under the following BioProject accession numbers: ASD dataset: PRJNA321909 (GSE81541- https://www.ncbi.nlm.nih.gov/bioproject/PRJNA321909) and PRJNA490887 (GSE119981- https://www.ncbi.nlm.nih.gov/bioproject/490887); Schizophrenia dataset: PRJNA421218 (https://www.ncbi.nlm.nih.gov/bioproject/PRJNA421218) and PRJNA422380 (https://www.ncbi.nlm.nih.gov/bioproject/PRJNA422380); and the multi-tissue dataset: PRJNA34535 (https://www.ncbi.nlm.nih.gov/bioproject/PRJNA34535).
