# Supplementary material for: Ribosomal DNA Copy Number Variation is Coupled with DNA Methylation Changes at the 45S rDNA Locus
Source: Epigenetics. 2023 Jun 27;18(1):2229203. doi: 10.1080/15592294.2023.2229203 (PMC10305490; doi:10.1080/15592294.2023.2229203)
Supplement: Supplemental Material [file KEPI_A_2229203_SM5210.zip › Supplementary files/Additional File 4.docx]

| **ID** | **Gender** | **Tissue** | **Age** | **Relative CN 18S** | **Relative CN 28S** | **rDNA Methylation** | **Source WGBS Libraries** |
| --- | --- | --- | --- | --- | --- | --- | --- |
| **STL001** | M | Adipose | 3 | 148.439 | 149.054 | 20.60% | Schultz et al. 2015 |
| **STL001** | M | Gastric | 3 | 156.057 | 152.882 | 29.80% | Schultz et al. 2015 |
| **STL001** | M | Lung | 3 | 141.858 | 147.586 | 27.80% | Schultz et al. 2015 |
| **STL001** | M | Psoas muscle | 3 | 136.374 | 135.252 | 27.20% | Schultz et al. 2015 |
| **STL001** | M | Bladder | 3 | 152.434 | 150.481 | 23.80% | Schultz et al. 2015 |
| **STL001** | M | Right ventricle | 3 | 148.794 | 146.718 | 21.90% | Schultz et al. 2015 |
| **STL001** | M | Spleen | 3 | 141.764 | 144.709 | 28.00% | Schultz et al. 2015 |
| **STL001** | M | Left ventricle | 3 | 141.715 | 132.513 | 21.80% | Schultz et al. 2015 |
| **STL001** | M | Small intestine | 3 | 142.932 | 145.684 | 27.90% | Schultz et al. 2015 |
| **STL002** | F | Adrenal gland | 30 | 168.744 | 172.763 | 30.30% | Schultz et al. 2015 |
| **STL002** | F | Aorta | 30 | 169.393 | 172.139 | 32.20% | Schultz et al. 2015 |
| **STL002** | F | Esophagus | 30 | 160.464 | 168.842 | 38.10% | Schultz et al. 2015 |
| **STL002** | F | Adipose | 30 | 165.103 | 165.258 | 34.40% | Schultz et al. 2015 |
| **STL002** | F | Gastric | 30 | 157.921 | 158.079 | 32.80% | Schultz et al. 2015 |
| **STL002** | F | Pancreas | 30 | 156.036 | 161.75 | 32.00% | Schultz et al. 2015 |
| **STL002** | F | Psoas muscle | 30 | 159.903 | 158.221 | 35.20% | Schultz et al. 2015 |
| **STL002** | F | Small intestine | 30 | 169.459 | 179.087 | 51.60% | Schultz et al. 2015 |
| **STL002** | F | Spleen | 30 | 165.584 | 165.396 | 32.80% | Schultz et al. 2015 |
| **STL002** | F | Ovary | 30 | 173.992 | 172.319 | 32.60% | Schultz et al. 2015 |
| **STL003** | M | Adrenal | 34 | 218.439 | 219.327 | 52.60% | Schultz et al. 2015 |
| **STL003** | M | Aorta | 34 | 258.285 | 263.197 | 55.50% | Schultz et al. 2015 |
| **STL003** | M | Adipose | 34 | 247.3863 | 256.601 | 58.20% | Schultz et al. 2015 |
| **STL003** | M | Right ventricle | 34 | 272.750 | 283.946 | 53.80% | Schultz et al. 2015 |
| **STL003** | M | Gastric | 34 | 245.969 | 254.078 | 60.80% | Schultz et al. 2015 |
| **STL003** | M | Right Atrium | 34 | 261.786 | 258.878 | 55.50% | Schultz et al. 2015 |
| **STL003** | M | Small Intestine | 34 | 227.508 | 254.463 | 71.00% | Schultz et al. 2015 |
