# Supplementary material for: Ribosomal DNA Copy Number Variation is Coupled with DNA Methylation Changes at the 45S rDNA Locus
Source: Epigenetics. 2023 Jun 27;18(1):2229203. doi: 10.1080/15592294.2023.2229203 (PMC10305490; doi:10.1080/15592294.2023.2229203)
Supplement: Supplemental Material [file KEPI_A_2229203_SM5210.zip › Supplementary files/Supplemental online material.docx]

**Supplemental online material.**

**Additional File 1. a)**  list of the samples used to test the correlation between rDNA copy number calculation via WGS and WGBS b) list of studied ASD brain samples. List of analyzed Schizohprenia and control c) neurons and d) oligodendrocytes.

**Additional File 2.** Correlation between relative 18S and 28S rDNA copy number in WGBS from a) Neurons and b) WGBS from Oligodendrocytes.

**Additional File 3.** Effect of read count on relative rDNA CN calculation and DNA methylation after subsequently decreasing the number of reads from original read count to 10% of original reads.

**Additional File 4.** Calculated relative 18S and 28S rDNA copy number and global rDNA methylation in several tissues from three individuals of 3, 30, and 34 years old.

**Additional File 5.** Relationship between 28S relative rDNA copy number and DNA methylation at the 45S rDNA locus in a) bulk brain tissue, c) neurons, and e) oligodendrocytes after correcting for age, gender and disease status. Dots indicate a 200 bp bin whose average DNA methylation was calculated. All p-values were FDR adjusted prior to plotting. The values above the dotted line (red dots) indicate a significant association (p < 0.05). The x-axes indicates the 45S rDNA coordinates. Scatter plots showing the relationship between 28S relative rDNA copy number and DNA methylation across bin with rDNA coordinates 7400-7599 in b) bulk brain tissue, d) neurons, and f) oligodendrocytes

**Additional File 6.** Relationship between relative rDNA Copy number and global DNA methylation of the 45S rDNA locus in multiple tissues in each of three individuals: A positive association is evident between relative rDNA copy number and average methylation in three post-mortem tissues (adipose, gastric, and small intestine) which were commonly analyzed in the three individuals.
