# Supplementary figures and images for: Ribosomal DNA Copy Number Variation is Coupled with DNA Methylation Changes at the 45S rDNA Locus
Source: Epigenetics. 2023 Jun 27;18(1):2229203. doi: 10.1080/15592294.2023.2229203 (PMC10305490; doi:10.1080/15592294.2023.2229203)

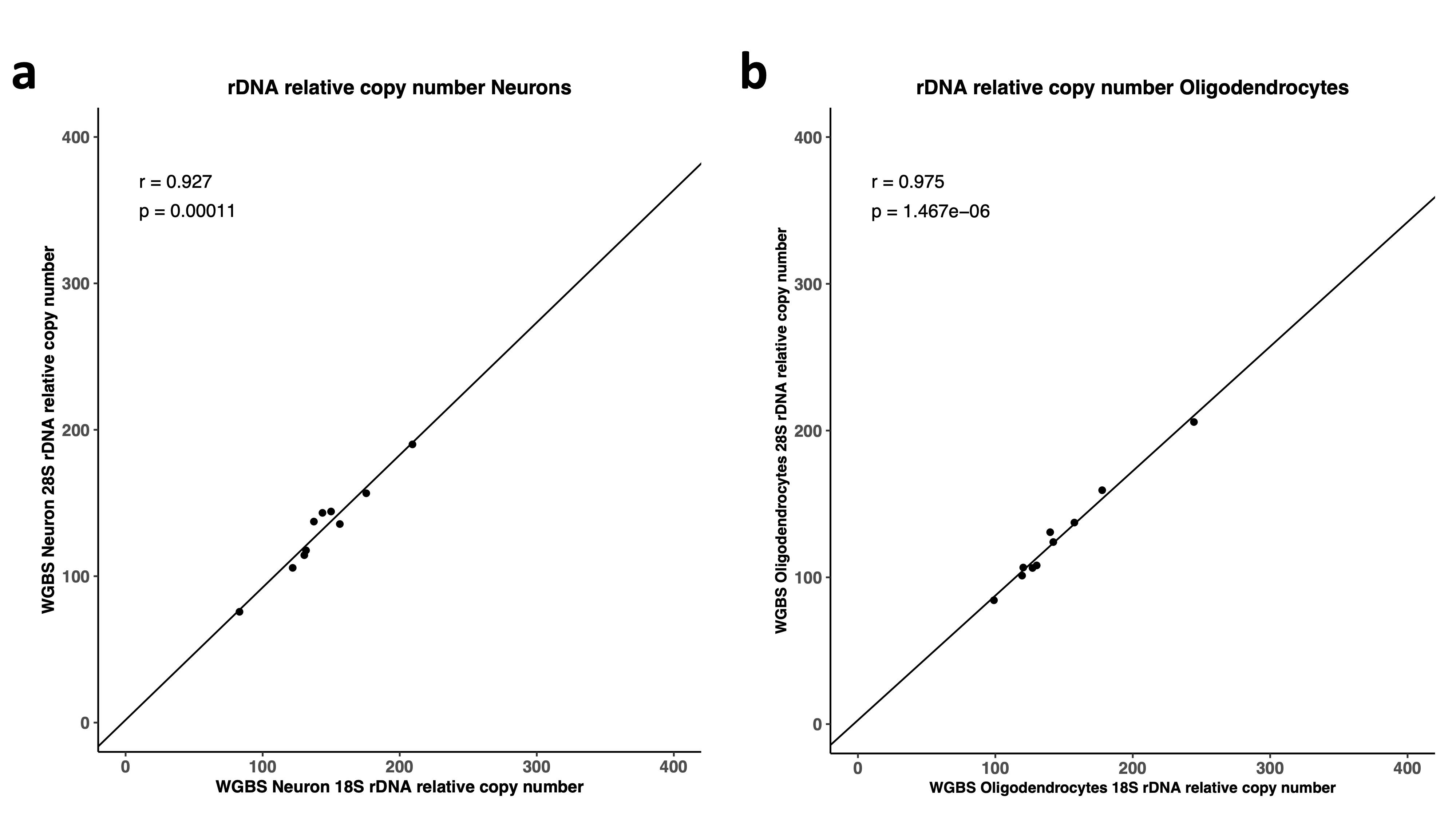

Supplement: Supplemental Material [file KEPI_A_2229203_SM5210.zip › Supplementary files/Additional File 2.tiff]

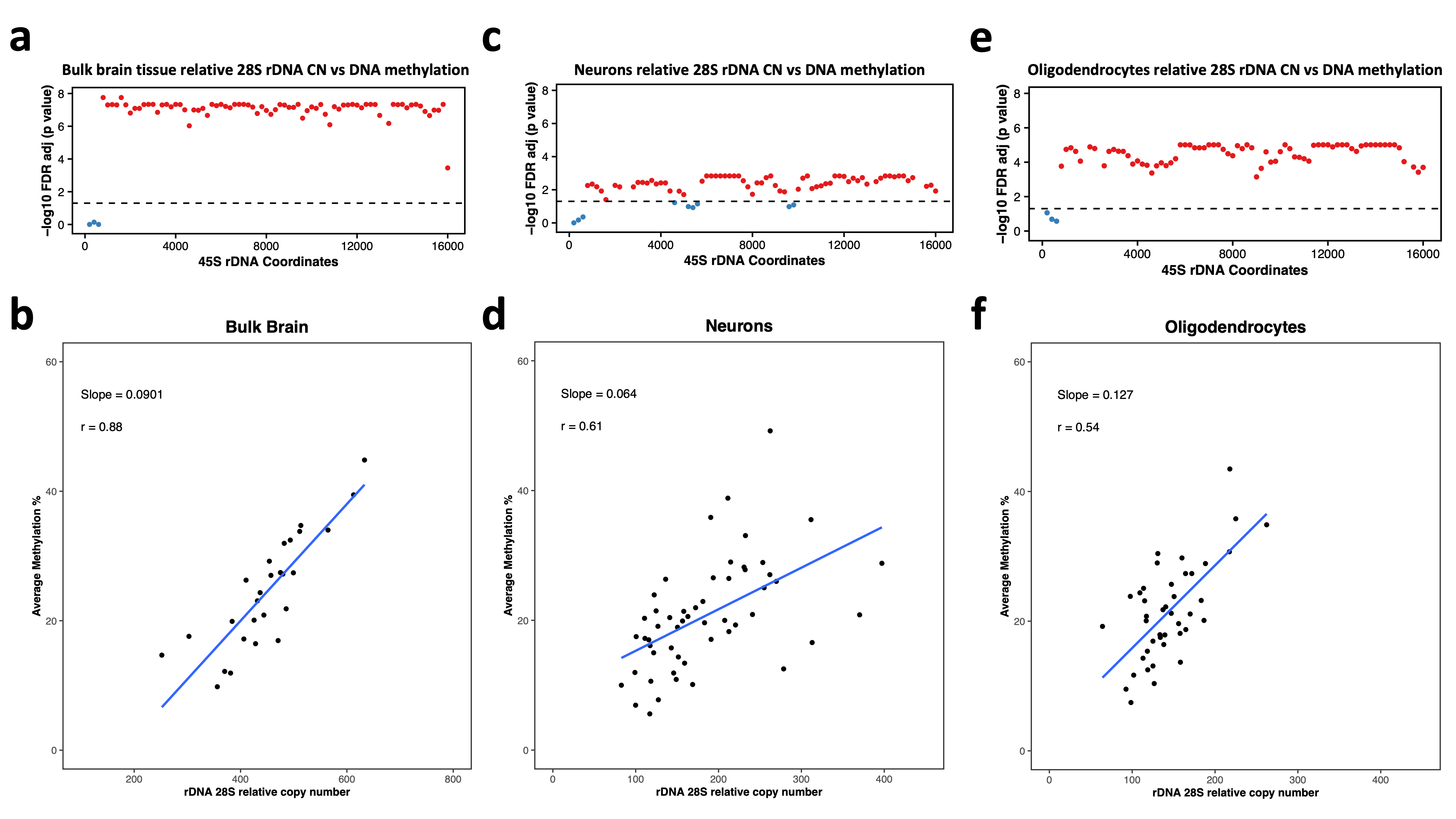

Supplement: Supplemental Material [file KEPI_A_2229203_SM5210.zip › Supplementary files/Additional File 5.tiff]

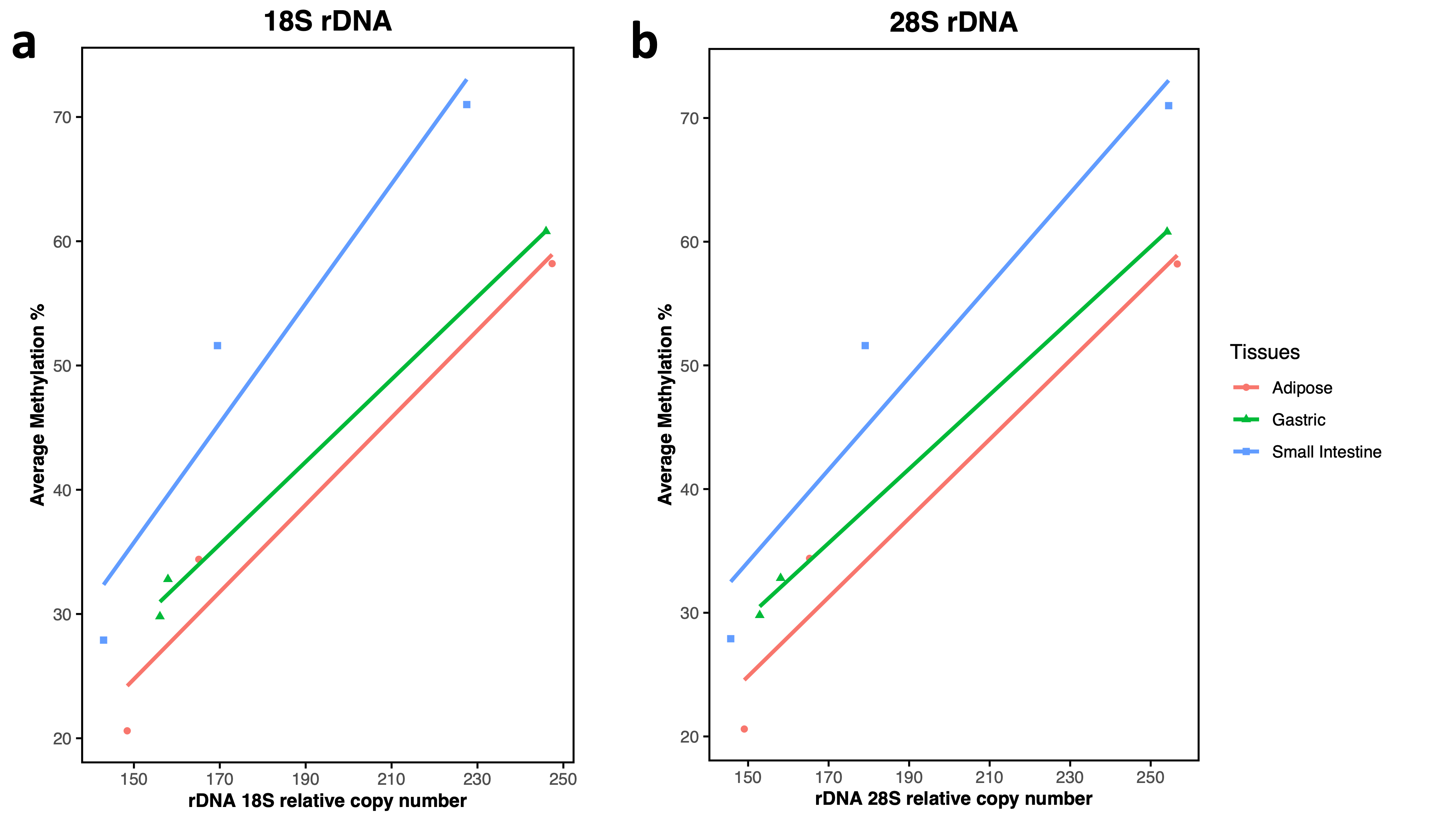

Supplement: Supplemental Material [file KEPI_A_2229203_SM5210.zip › Supplementary files/Additional File 6.tiff]
